# Management of brain metastases in older adults with non–small cell lung cancer

**DOI:** 10.3389/fonc.2026.1858724

**Published:** 2026-07-28

**Authors:** Julia Belone Lopes, Daniel Schep, Arjun Sahgal, Rodrigo F. Rodrigues, Ines B. Menjak

**Affiliations:** 1 Division of Medical Oncology, Department of Medicine, Sunnybrook Health Sciences Centre, University of Toronto, Toronto, ON, Canada; 2 Division of Radiation Oncology, Department of Medicine, Sunnybrook Health Sciences Centre, University of Toronto, Toronto, ON, Canada; 3 Division of Neurosurgery, Department of Surgery, St. Michael’s Hospital, University of Toronto, Toronto, ON, Canada

**Keywords:** brain metastases, geriatric oncology, lung cancer, NSCLC, older adults

## Abstract

Brain metastases (BrM) are a major cause of morbidity in patients with non–small-cell lung cancer (NSCLC). As global populations age and systemic therapies improve survival, the number of older individuals presenting with BrM continues to rise. However, older adults remain underrepresented in clinical trials. Thus, treatment decisions in this population are often based on limited evidence from randomized prospective studies. Beyond chronological age, older adults with cancer frequently present with multiple comorbidities, frailty, baseline cognitive impairment, and reduced physiologic reserve. These factors may contribute to suboptimal treatment in clinical practice and highlight that age alone is an insufficient surrogate for vulnerability, underscoring the need for individualized geriatric assessment. In this review, we summarize the available evidence for CNS-directed and systemic therapies in older patients with NSCLC and BrM, discuss the options for local and systemic treatment strategies, and examine how geriatric assessment tools can inform individualized treatment decisions in this underrepresented population.

## Introduction

Brain metastases (BrM) are a major cause of morbidity in patients with non–small-cell lung cancer (NSCLC). The prevalence of BrM as first presentation of disease is 15%-20%, and up to 40% eventually develop intracranial disease over the course of illness (1). These figures can be even higher in tumors harboring specific oncogenic drivers, such as epidermal growth factor receptor (EGFR) mutations or anaplastic lymphoma kinase (ALK) gene rearrangements (2). As global populations age and systemic therapies improve survival, the number of older individuals presenting with BrM continues to rise. However, older adults remain underrepresented in clinical trials (3, 4), thus, treatment decisions in this population are often based on limited evidence from randomized prospective studies.

There is no single accepted age cut-off for defining a geriatric population in oncology. The National Comprehensive Cancer Network (NCCN) Geriatric Oncology Guidelines categorize older adults as young-old (65–75 years), old (76–85 years), and oldest-old (>85 years) (5). It is important to note that approximately 72% of new lung cancer diagnoses occur in individuals older than 65 years, while those older than 75 years account for approximately 37% of new diagnoses (6).

Age is a well-recognized poor prognostic factor in NSCLC patients with BrM, SEER (2010–2021) reported an overall median survival of 5.0 months for NSCLC BrM across all ages. Notably, patients aged <65 years had a median overall survival (mOS) of 7.0 months, while those aged ≥65 years had a mOS of only 4.0 months (7). In contrast, outcomes have substantially improved with the advent of immunotherapy, in a pooled analysis of KEYNOTE-021, KEYNOTE-189, and KEYNOTE-407, patients with stable BrM treated with pembrolizumab plus platinum-based chemotherapy achieved a mOS of 18.8 months versus 7.6 months with chemotherapy alone (HR 0.48) (8). These outcomes are even more favorable for patients harboring actionable oncogenic drivers. In the FLAURA2 trial, among patients with baseline central nervous system (CNS) metastases, 36-month overall survival was 57% with osimertinib plus chemotherapy compared to 40% with osimertinib monotherapy (9). In the CROWN trial, first-line lorlatinib in ALK-positive NSCLC achieved unprecedented outcomes; among patients with baseline BrM, 50% were alive without progression at 3 years, and the probability of remaining free from intracranial progression was 83% in this subgroup and 92% in the intention to treat (ITT) population at 5 years (10, 11). However, the study population was relatively young (median age 59 years; range 26–90), with only 35% of patients aged ≥65 years, reflecting the typical epidemiology of ALK-positive NSCLC.

**Figure 1:**
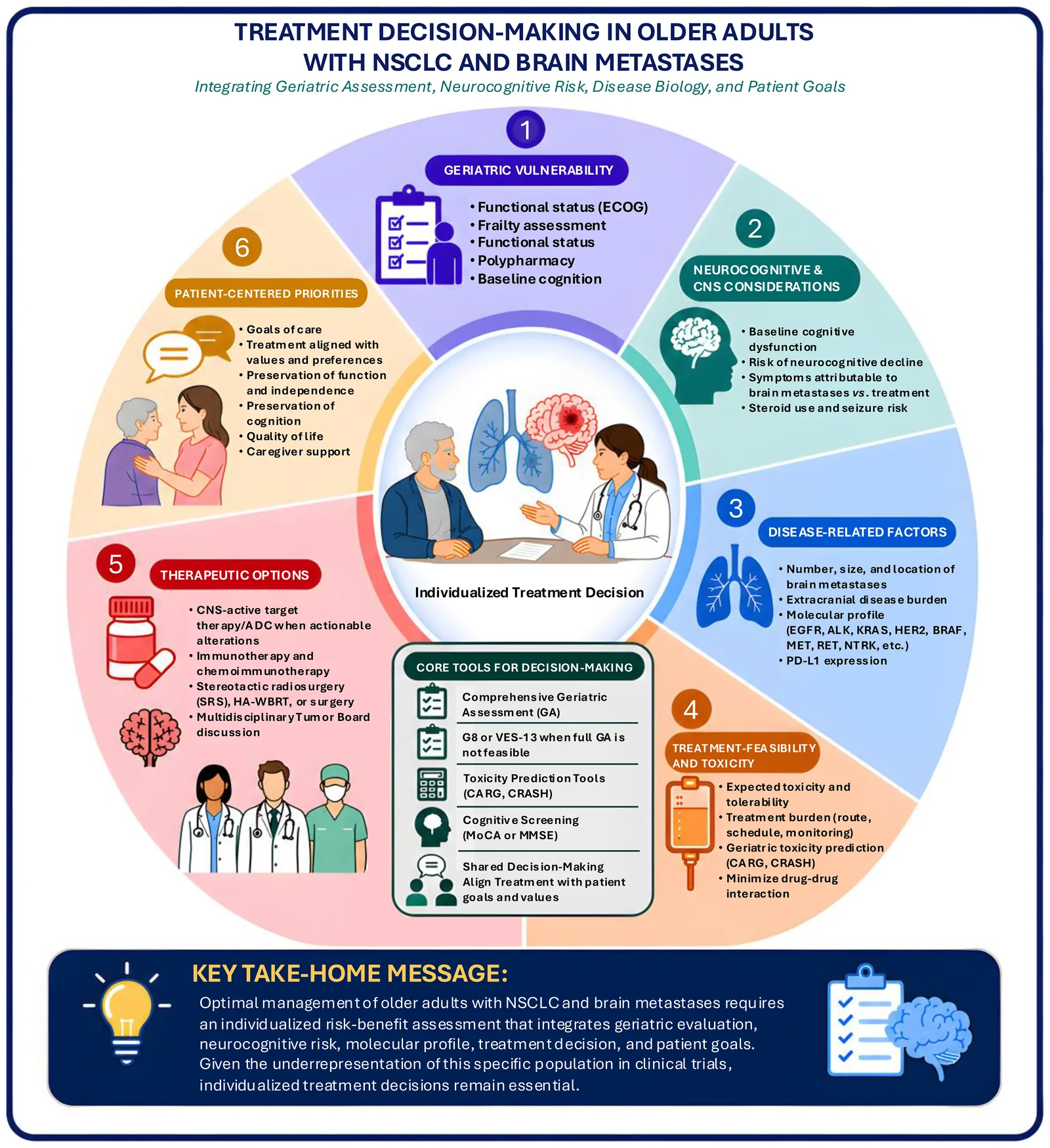
Proposed patient-friendly framework for treatment decision-making in older adults with NSCLC and brain metastases.

Beyond chronological age, older adults with cancer frequently present with multiple comorbidities, frailty, baseline cognitive impairment, and reduced physiologic reserve (12). These factors may contribute to suboptimal treatment in clinical practice and highlight that age alone is an insufficient surrogate for vulnerability, underscoring the need for individualized geriatric assessment (see **Figure 1**) (4, 13).

In this review, we summarize the available evidence for CNS-directed and systemic therapies in older patients with NSCLC and BrM, discuss the options for local and systemic treatment strategies, and examine how geriatric assessment tools can inform individualized treatment decisions in this underrepresented population.

## Presentation and prognostic assessment in older patients with brain metastases

Across several solid tumors, approximately 26% of patients demonstrate pre-treatment cognitive impairment when assessed with screening tools (14). This challenge is substantially amplified in patients with BrM, where neurocognitive dysfunction is highly prevalent, with almost 80% of patients showing objective deficits in at least one domain prior to radiotherapy, most frequently memory, processing speed, psychomotor speed, and social cognition (15). In addition, approximately 70% report subjective cognitive decline, most often in attention, memory, and thinking, though the majority reported stable functioning across most individual domains (15). Beyond cognitive effects, brain metastases can present with headaches, fatigue, motor weakness, coordination deficits, and seizures. Screening tools such as the Mini-Mental State Examination (MMSE) and the Montreal Cognitive Assessment (MoCA) are widely used due to their brevity and ease of administration, however, the MMSE lacks sensitivity for detecting deficits in memory, executive function, and psychomotor speed in patients with brain tumors (16). MoCA seems to be more sensitive than the MMSE and is quick to administer (about 10 min), though both instruments have limited discriminative accuracy compared with formal neuropsychological testing (16).

Several other prognostic indices have been developed to estimate survival in patients with BrM and guide treatment decisions. One of the earliest models was the Recursive Partitioning Analysis (RPA), developed by the radiation therapy oncology group (RTOG) in 1997, which incorporates patient age, Karnofsky Performance Status (KPS), control of the primary tumor, and the presence of extracranial metastases (17). Subsequent models expanded on these variables: the Score Index for Radiosurgery (SIR) added the number of brain metastases and volume of the largest lesion (18), while the Basic Score for Brain Metastases (BSBM) offered a simplified framework based on KPS, primary tumor control, and extracranial disease status (19). To improve stratification in patients aged ≥65, the Elderly-Specific BSBM (ES-BSBM) further incorporated brain metastasis specific factors, including lesion number and cumulative tumor volume (20).

In 2008, Sperduto and colleagues introduced the Graded Prognostic Assessment for Patients With Lung Cancer and Brain Metastases (Lung-GPA), integrating age, KPS, number of BrM, and extracranial disease (21). This framework evolved through successive refinements, the Lung-molGPA (2017) incorporated molecular alterations such as EGFR mutations and ALK rearrangements, and a subsequent update version (2022) added PD-L1 expression as a prognostic factor (22, 23).

Comparative analyses have demonstrated that the Lung-molGPA achieves superior predictive accuracy compared with RPA, GPA, BSBM, and DS-GPA in predicting outcomes for NSCLC patients with BrM (24). These prognostic tools are particularly valuable in older adults, as they help clinicians balance expected survival, treatment intensity, and preservation of functional status when selecting individualized therapeutic strategies.

## Geriatric assessment and toxicity prediction

Comprehensive geriatric assessment (GA) is recommended by the ASCO Geriatric Oncology Guideline for all older patients (≥65 years) with cancer (25). GA evaluates multiple domains, including function, comorbidity, falls, cognition, nutrition, and social support, and can predict treatment toxicity, estimate life expectancy, and identify reversible vulnerabilities not captured by standard oncology assessments (25, 26). The landmark GAP70+ trial demonstrated that GA-guided management reduced grade 3–5 toxicity from 71% to 51% (RR 0.74), notably, more patients in the intervention group received upfront dose reductions without compromising OS (27).

Although GA is the gold standard for identifying frail older patients, traditional versions requiring proctored assessments can take 30–60 minutes and are often not feasible in many busy oncology practices without dedicated resources (25). To address this, abbreviated GA tools have been developed, such as the Practical Geriatric Assessment (PGA), which takes 10–25 minutes and can be completed by the patient or caregiver before the visit or in the waiting area, minimizing required staff time. The ongoing PACE-70 study is evaluating PGA implementation and its impact on chemotherapy dose modification among older adults with advanced cancer in community oncology settings (28). In addition, validated screening tools such as the Geriatric 8 (G8), originally designed for patients older than 70 years and administered by a healthcare professional in approximately 4 minutes, and the Vulnerable Elders Survey-13 (VES-13), a self-administered questionnaire that takes approximately 5 minutes, are practical options for busy clinical settings. Both have been validated in oncology populations to predict toxicity, hospitalization, and mortality, with the G8 demonstrating higher sensitivity and the VES-13 higher specificity for detecting geriatric impairments warranting further assessment (29). These screening tools do not replace a comprehensive GA but are recommended to identify patients who may benefit from a full assessment followed by geriatric intervention when indicated.

Beyond comprehensive geriatric assessment, optimal care of older adults with BrM requires a multidisciplinary approach. Early palliative care integration has consistently demonstrated improvements in quality of life and symptom burden, with a possible OS benefit (30, 31). In addition, dedicated multidisciplinary BrM programs have been associated with improved treatment planning and survival outcomes (32). Rehabilitation strategies through occupational therapy, physiotherapy, speech therapy, and neuropsychological interventions are important components of comprehensive geriatric oncology care. In parallel, regular evaluation of caregiver burden and support needs should be also incorporated into clinical care (5).

Several validated tools have also been developed to guide treatment decisions and minimize toxicity in older adults, including models that predict chemotherapy-related toxicity. The Cancer and Aging Research Group (CARG) tool was originally developed and validated in older adults with solid tumors (n=500; mean age 73 years), with lung cancer being the most represented tumor type (29%). It includes 11 variables including cancer and treatment-related factors (age, cancer type, planned chemotherapy dosing, number of chemotherapy drugs), patient-reported geriatric domains (hearing, falls, walking ability, ability to take medications, social activity limitation), and laboratory values (hemoglobin and creatinine clearance). It stratifies patients into low (0–5 points, ~30% risk), intermediate (6–9 points, ~52% risk), and high risk (10–19 points, ~83% risk) of grade ≥3 toxicities (33). A disease-specific module (CARG-BC) has been developed and externally validated for breast cancer, but no lung cancer-specific version currently exists (34).

The Chemotherapy Risk Assessment Scale for High-Age Patients (CRASH) score is an alternative tool that incorporates the specific chemotherapy regimen, laboratory values (LDH, diastolic blood pressure, hemoglobin, creatinine clearance, albumin), and functional (ECOG performance status), nutritional (Mini Nutritional Assessment), and cognitive (MMSE) assessments (35). It predicts hematological (grade 4) and non-hematological (grade 3–4) toxicities separately, with risk ranging from 7% to 100% for hematological and 33% to 93% for non-hematological toxicity across categories (35). Both CARG and CRASH have demonstrated predictive value in their original cohorts, though external validation studies have shown variable performance across different populations (36).

## Radiotherapy approaches: shifting from WBRT to SRS

Radiation is the backbone of treatment for BrM, either alone or in conjunction with surgical resection. The technological evolution in radiation oncology, allowing for precision focal radiation with tumor ablative doses, has shifted practice away from routine whole brain radiation therapy (WBRT) to stereotactic radiosurgery (SRS) (37). Better neurocognitive and quality-of-life (QOL) outcomes have been observed in several randomized trials both for intact metastases (including small cell lung cancer (38)) and post-operative surgical cavities (39) following SRS alone, despite the benefit of reducing the risk of distant brain failure associated with upfront WBRT (40–44). These Phase 3 randomized trials typically enrolled patients presenting with up to 4 metastases; however, a recent randomized trial allowing patients with up to 20 metastases proved superior functional outcomes following SRS (45). The trend to evaluate SRS alone in patients with multiple metastases is also apparent in a study now closed to accrual (Clinical Trials number NCT03775330) that allowed patients with up to 30 metastases, and on the day of treatment up to 50 metastases, to be treated with SRS. This is of considerable importance for the elderly, as the intent of any brain-directed treatment should be to preserve cognition and quality-of-life given the worse overall prognoses associated with increasing age. Therefore, if an older patient with up to 20 metastases is eligible for SRS the current evidence would support SRS alone as first-line, as long as the expected prognosis is at least 3 months which is typically determined according to performance status (PS).

Should new BrM develop after prior treatment with SRS, then further courses of SRS can be considered, which has been shown to defer the need for WBRT in selected patients (46). At the time of CNS-only oligoprogression, discussion of SRS versus a switch in systemic therapy could be considered depending on the subsequent agent’s CNS penetrance and the volume of disease. WBRT may still be considered for older patients if presenting with a significant burden of intra-cranial disease (>20 metastases) or had received prior SRS such that further SRS is not indicated or possible. In these situations, the aim should be to deliver hippocampal avoidance WBRT (HA-WBRT), as it has been shown in randomized trials to reduce the detrimental neurocognitive impact associated with traditional WBRT (47, 48). However, it is not a replacement for SRS (45), and HA-WBRT is not indicated in the presence of leptomeningeal disease. The use of adjunctive pharmacologic therapies such as memantine (49) or donepezil (50) can be considered, however, the evidence is not clear as to the benefit and older patients are often taking multiple medications such that drug interactions must be considered.

One emerging attractive option for patients with an actionable mutation and eligible for a CNS penetrant systemic agent is to defer SRS and/or WBRT, and treat with drug alone (51). Several agents have demonstrated activity with prolonged intracranial PFS, particularly in those with ALK-positive and EGFR-mutant NSCLC (52–54). In this setting, SRS may be reserved as a salvage treatment for nonresponding metastases, or in the context of oligoprogression. However, we are learning that these agents may not be sufficient for larger metastases, sub-optimal in the setting of prior radiation, sub-type of the mutation may matter and, therefore, the sequence of therapy requires thoughtful consideration and clinical trials to optimize the approach in this new era of CNS active targeted therapies (55, 56).

The role of surgery for large metastases (3–4 cm) is a matter of multidisciplinary discussion if minimally symptomatic, as hypofractionated SRS has been shown to be effective with respect to local control while maintaining a reasonable risk of radiation necrosis (57, 58). Moreover, sequencing a CNS penetrant systemic agent with SRS may allow for a synergistic response to optimize tumor shrinkage. This potential has resulted in a resurgence of what has been considered “staged SRS” (59), such that treatment with a CNS-active drug is combined with SRS fractions delivered in-between drug cycles rather than all at once (60). For example, instead of delivering 30 Gy in 5 daily fractions as per standard practice for large brain metastases (>2cm), each fraction of 6 Gy is delivered every 2–3 weeks while holding the agent 1–2 days before and after SRS (57) or on the off weeks for systemic agents administered on a q2–3 week cycle. This approach avoids delays in starting the drug and allows sequential, fraction-to-fraction, adaptation of the SRS volume on what is hopefully a shrinking metastasis. Ultimately, the aim is to reduce the risk of radiation necrosis and need for surgery, particularly in older patients.

## The role of neurosurgery

Surgical resection of BrM is considered in extreme presentations of symptomatic mass effect, hydrocephalus, metastases >4 cm or toxic edema, and is typically followed by adjuvant SRS to the resection cavity (61). In older patients, contemporary evidence supports the feasibility of neurosurgical intervention in selected patients. A propensity score-matched analysis found that age ≥75 years did not increase 30-day mortality or major complications when controlled for other risk factors, and a matched-cohort study of octogenarians showed no significant differences in postoperative outcomes compared to younger patients (62, 63). Critically, frailty rather than chronological age appears to be the dominant predictor of surgical outcomes, a national database analysis of 13,650 patients demonstrated that the modified frailty index was a stronger predictor of postoperative mortality than age alone (64). These data reinforce that surgical candidacy in older patients should be carefully evaluated case-by-case in a multidisciplinary setting and guided by a comprehensive GA. When feasible, formal baseline neuropsychological testing should also be considered to establish a cognitive reference point, given the high prevalence of pre-existing neurocognitive impairment in this population and the potential impact of both surgery and adjuvant radiation on cognitive function. Lastly, the role of surgery performed solely to obtain tissue for molecular profiling should likewise be discussed carefully in a multidisciplinary forum, particularly in light of the growing availability of circulating tumor DNA (ctDNA) as a tool for molecular diagnosis (65). Following surgery, post-operative cavity radiation has been shown to reduce the risk of neuro-cognitive decline. However, increasing evidence supports the use of pre-operative SRS in select cases to reduce the risk of radiation necrosis and leptomeningeal dissemination (66, 67) and comparative randomized trials are ongoing.

## Combined local and systemic treatment strategies

An important decision in older patients with oncogene-driven NSCLC and available CNS-penetrant targeted agents is when local treatment can safely be deferred in favor of upfront systemic therapy, aiming to minimize toxicities. This question was discussed in the radiation section above and currently remains an active area of investigation, without a unique consensus.

Most pivotal trials excluded patients with active BrM, so this decision relies largely on small prospective trials and real-world studies. In a large real-world cohort from Italy, including 177 NSCLC patients with synchronous BrM receiving first-line TKIs or pembrolizumab (119 received systemic therapy alone and 58 received combined systemic therapy and advanced RT). The radiation-omission group was predominantly older, with over half aged 70 or above. Despite this, the addition of RT was associated with significantly better OS (22.6 vs. 12.1 months) and PFS (14.8 vs. 9.4 months), with no age-stratified analysis reported (68). In another retrospective study from the UK including 453 NSCLC patients with BrM and a median age of 69 years, only 5.4% harbored actionable mutations and 11.5% were PD-L1 positive, reflecting a predominantly unselected population. Approximately one-third received local treatment with surgery or radiation, with a best intracranial response rate of 44%. Among those treated with systemic anticancer therapy, the best intracranial response rate was 35%. Performance status was the strongest predictor of survival (5.6 vs. 1.4 months for good vs. poor PS), and driver mutation positivity was associated with modestly better outcomes (69).

The first randomized evidence on SRS deferral in the EGFR setting came from the STARLET trial, which randomized 79 patients with EGFR-mutant NSCLC (median age 67 years) and up to 10 minimally symptomatic BrM to upfront SRS followed by osimertinib or osimertinib monotherapy. The 12-month intracranial PFS was not significantly different between arms (difference 11%, p = 0.31; median ic-PFS 21.9 vs. 17.2 months), and while median OS was numerically longer with the combined approach (46.1 vs. 29.1 months), the trial was not powered for this endpoint. Among patients with intracranial progression, 57% in the monotherapy arm ultimately required salvage SRS compared to 35% in the combined arm, suggesting that deferral may delay rather than eliminate the need for local treatment. The most common adverse events in the combination arm were fatigue (34%), headache (26%), and radionecrosis (21%); neurocognitive outcomes are pending publication (52).

The growing body of evidence suggests that radiation deferral will become more common as small molecules with high CNS activity continue to be developed (70). Current ASCO-SNO-ASTRO guidelines support deferring local treatment in asymptomatic brain metastases patients receiving CNS-penetrant systemic therapy for EGFR-mutant or ALK-rearranged NSCLC. While there is evidence of intracranial activity with chemo-immunotherapy combinations such as those from KEYNOTE-189, guidelines do not currently support deferring local treatment in that setting (61). The rapid development of potent CNS-active agents across other driver mutations (described in detail below) is outpacing guideline recommendations, and treatment sequencing decisions will likely require ongoing individualized reassessment within a multidisciplinary team, particularly in older patients, where toxicity and quality of life carry added weight. When local therapy is deferred, close monitoring with brain MRI is essential to ensure timely intervention at the first sign of intracranial progression.

## Chemoimmunotherapy: CNS activity and considerations in older adults

For patients with advanced NSCLC without actionable oncogenic drivers, chemotherapy combined with immune checkpoint inhibitors (ICIs) has become the standard first-line treatment, investigated in several pivotal trials regardless of PD-L1 expression. The main chemoimmunotherapy regimens supported by phase III evidence in this setting include pembrolizumab plus platinum-pemetrexed for non-squamous (KEYNOTE-189) or platinum-taxane for squamous histology (KEYNOTE-407), atezolizumab plus carboplatin-paclitaxel-bevacizumab or carboplatin-nab-paclitaxel (IMpower150, IMpower130), cemiplimab plus platinum-doublet chemotherapy (EMPOWER-Lung 3), nivolumab plus ipilimumab with two cycles of platinum-doublet chemotherapy (CheckMate 9LA), and tremelimumab plus durvalumab combined with platinum-based chemotherapy (POSEIDON) (71–77). However, patients with BrM and older adults, two populations that frequently overlap in clinical practice, have been historically underrepresented in these landmark studies, raising questions about the generalizability of their results (4).

## Efficacy of chemoimmunotherapy in patients with brain metastases

Across the pivotal chemoimmunotherapy trials mentioned above, patients with treated and stable or asymptomatic BrM were generally eligible but represented a small proportion of enrolled populations. In KEYNOTE-189 approximately 17% of patients had baseline BrM, and exploratory analyses suggested preserved OS and PFS benefit in this subgroup (78). A pooled analysis of KEYNOTE-021G, -189, and -407 assessed 171 patients with treated or untreated stable BrM (median age 63 years; range 35–82). Notably, 38% of this subgroup had PD-L1 TPS <1%. Pembrolizumab plus chemotherapy improved OS (18.8 vs. 7.6 months; HR 0.48) and PFS (6.9 vs. 4.1 months; HR 0.44) regardless of PD-L1 status, although in a subgroup analysis the OS benefit did not reach statistical significance among patients with PD-L1 TPS 1–49%. No age-specific subgroup analysis was performed within this cohort (9).

In IMpower150 and IMpower 130, patients with treated and stable BrM were allowed, though no dedicated subgroup analysis of OS or PFS in patients with baseline BrM was reported (73, 74). Notably, 8.3% of the ITT population (100 patients) developed new brain metastases during follow-up, with a trend toward delayed development of new CNS lesions with atezolizumab plus bevacizumab plus carboplatin and paclitaxel (ABCP) versus bevacizumab plus carboplatin and paclitaxel (BCP) (HR 0.68; 95% CI 0.39–1.19), although this was not formally tested (79). A pooled analysis of individual patient data from IMpower130, IMpower131, and IMpower150 (2,380 patients without baseline BrM and without EGFR/ALK mutations) showed that adding atezolizumab to chemotherapy did not significantly reduce the cumulative incidence of new BrM (p = .888), however, on multivariate analysis, the use of bevacizumab was independently associated with a reduced risk of developing BrM (80). In EMPOWER-Lung 3, approximately 7% of patients had treated and stable BrM at baseline. At the 2-year follow-up, the BrM subgroup showed a consistent OS benefit favoring the combination, the PFS analysis in this subgroup did not reach statistical significance, however, this was an exploratory analysis with limited sample size (81).

In CheckMate 9LA (N = 719), median age was 65 years, with 41% of patients aged 65–75 and 10% aged >75 years; 18% had treated and stable BrM at baseline in the experimental arm. At the 6-year update, the OS benefit with nivolumab plus ipilimumab plus chemotherapy was preserved in the ITT population (mOS 15.6 vs. 11 months), and this benefit was consistent across nearly all subgroups, with the exception of patients aged >75 years and never smokers, notably, no significant benefit was observed even in the 65–75 age subgroup. Among patients with baseline BrM, intracranial PFS was 11.4 vs 4.6 months favoring the combination (HR 0.42). However, in the ITT population, this benefit was associated with increased toxicity, with grade ≥3 treatment-related adverse events occurring in 48% versus 38% (82). The prospective phase II NIke trial further evaluated this regimen in 30 patients with untreated brain metastases (median age 66.5 years), reporting an intracranial response rate of 50% (83).

In the POSEIDON trial (N = 1,013; median age 63 years in the experimental arm, with approximately 10% having stable and treated BrM), tremelimumab plus durvalumab plus chemotherapy significantly improved OS versus chemotherapy alone (HR 0.76; 5-year OS rate 15.7% vs. 6.8%). Subgroup analyses showed an OS benefit in patients aged ≥65 years, but this was not statistically significant in those with baseline BrM and never smokers. The most pronounced benefit was observed in the non-squamous cell histology subgroup and among patients harboring biomarkers associated with poor prognosis (STK11, KEAP1 and KRAS mutations). Grade 3–4 adverse events occurred in 51.8% versus 44.4% in the chemotherapy control arm (76, 84). A reconstructed individual patient data meta-analysis including 2,881 patients compared dual checkpoint blockade (anti–PD-1/PD-L1 plus anti–CTLA-4) with anti–PD-1/PD-L1 monotherapy. No significant difference in OS was observed across histological subtypes. Greater benefit with dual blockade was seen in patients with STK11 mutations (HR 0.67; p = 0.012) and PD-L1 TPS <1% (HR 0.85; p = 0.021). However, no age-stratified or BrM analyses were performed (85).

Beyond these pivotal trials, the prospective single-arm phase II Atezo-Brain study specifically evaluated atezolizumab plus carboplatin and pemetrexed in 40 patients with advanced nonsquamous NSCLC and untreated brain metastases (median age 62 years). The intracranial overall response rate (iORR) was 42.7%, with a median intracranial PFS (iPFS) of 6.9 months, a systemic median PFS of 8.9 months, and median OS of 11.8 months, corresponding to a 2-year OS rate 27.5%. Notably, responses were observed regardless of PD-L1 expression, and 55% of patients were receiving dexamethasone at enrollment without apparent detriment to efficacy (86).

Real-world data have provided valuable insights into treatment outcomes in older patients with NSCLC and brain metastases. A retrospective cohort from Serbia (approximately 40% aged ≥65 years) identified age <65 years, single brain metastasis, timing of metastasis diagnosis, and combined local plus systemic therapy as independent predictors of improved OS, underscoring the prognostic impact of age and treatment access in routine clinical practice (87). In the largest retrospective real-world study specifically focused on older patients with NSCLC and synchronous brain metastases (1,303 received chemotherapy, 178 received ICI; median age 71 years), ICI-treated patients received either monotherapy (22%) or combination chemo-immunotherapy, most commonly pembrolizumab-based regimens. PD-L1 status was unavailable in this database, and all patients had treated BrM. ICI-based combination therapy was associated with better OS and a lower risk of death compared to chemotherapy alone, with the survival benefit emerging approximately 6 months after treatment initiation (88).

These data collectively support the intracranial activity of chemo-immunotherapy combinations across multiple ICI-based regimens, and real-world evidence further supports their efficacy in the older population.

## Efficacy and safety of chemoimmunotherapy in older adults

In the pivotal trials, patients aged ≥65 years typically comprised approximately 50% of enrolled populations, and subgroup analyses generally showed preserved benefit. In KEYNOTE-189, the OS benefit was maintained in patients aged ≥65 years (HR 0.64), although PFS did not reach statistical significance (71). In KEYNOTE-407, patients aged ≥65 years showed numerically favorable trends in PFS and OS, although just PFS reached statistical significance (72). Importantly, none of these trials reported specific survival data for patients aged ≥75 years, who represented only 9% of participants in KEYNOTE-189 (89).

The evidence for patients aged ≥75 years comes primarily from pooled analyses, meta-analyses, and real-world studies. A Cochrane systematic review (17 trials; 4,276 patients aged ≥65 years) demonstrated that the addition of ICIs to platinum-based chemotherapy improved OS (HR 0.78) and PFS (HR 0.61) compared with chemotherapy alone. However, among patients aged ≥75 years, there may be no difference in OS and PFS, though this represents low-certainty evidence given the small sample size (n=297) (90). A large retrospective cohort study from 58 centers in Japan assessed 1,245 patients aged ≥75 years (median age 78 years; range 75–95) with advanced NSCLC, 13% with baseline BrM. After propensity score matching, no significant differences in OS or PFS were found between ICI-chemotherapy and ICI monotherapy, regardless of PD-L1 expression (TPS 1–49% or ≥50%). Subgroup analyses by histology showed divergent, although non-significant, trends, among patients with adenocarcinoma, ICI monotherapy was associated with numerically longer OS than ICI-chemotherapy (26.2 vs 20.4 months), whereas in squamous cell carcinoma there was a numerical trend in favor of combination therapy (18.3 vs 13.3 months). From a safety standpoint, grade ≥3 immune-related adverse events were significantly more frequent with ICI-chemotherapy than ICI alone (24.3% vs. 17.9%), suggesting that adding chemotherapy may increase toxicity without providing a clear survival benefit in this older population, although this finding should be interpreted cautiously given the retrospective design and the exclusively Japanese multicenter cohort (89).

In the context of dual immune checkpoint blockade in older patients, the ENERGY trial (GFPC 08-2015) provides the only phase III, which enrolled 204 patients aged ≥70 years with ECOG PS 0–2 or aged <70 years with ECOG PS 2 (median age 74 years; 59% PD-L1 <1% and only 3% PD-L1 ≥50%), randomized to receive nivolumab plus ipilimumab (N+I) versus carboplatin-based chemotherapy. Notably, the control arm did not include immunotherapy, likely reflecting the study’s design and approval timeline. N+I did not significantly improve OS over chemotherapy in the overall population. However, in fit elderly patients (PS 0–1; median age 76 years), a significant OS benefit was observed with I+N (22.6 vs. 11.8 months), whereas the effect appeared detrimental in patients with PS 2 (2.9 vs. 6.1 months; HR 1.32). Grade ≥3 treatment-related serious adverse events were lower with N+I than with chemotherapy (31.4% vs. 49.5%), though treatment discontinuation due to toxicity was higher (28.6% vs. 22.3%). These results reinforce that PS rather than chronological age is the critical determinant of benefit from immunotherapy (91).

An Italian real-world study (Real-Combo Lung) comparing older (≥75 years; n=89) versus younger patients receiving first-line chemoimmunotherapy found no significant differences in PFS or OS. Rather than age, ECOG PS ≥2 and baseline corticosteroid use were identified as the main adverse prognostic factors (92). Taken together, these findings raise the question of whether adding chemotherapy to immunotherapy provides meaningful benefit in patients aged ≥75 years and support an individualized approach based on functional status and comorbidities rather than chronological age alone.

Beyond efficacy, treatment-related toxicities are a critical consideration in this population and may be more pronounced given the high prevalence of baseline cognitive impairment and reduced physiologic reserve in older adults. Chemotherapy-related cognitive impairment (commonly known as “chemobrain” or “chemofog”) has been reported in approximately 32.8% of older patients with lung cancer. Fatigue, anxiety, depression, and low cognitive reserve are recognized risk factors (93). A large FDA review of patients treated with ICIs showed higher rates of immune-related adverse events (irAEs) among patients aged 65–74 and 75–85 years compared with younger patients. Cardiovascular, renal, and musculoskeletal irAEs were more common in older age groups, while no major differences were observed in cutaneous, neurological, or hematological toxicities (94). Consistent with these findings, another study reported higher rates of hospitalization, treatment discontinuation due to irAEs, and use of corticosteroids or other immunosuppressive therapies among older patients (95).

## ICI monotherapy in older adults with high PD-L1 expression

For patients with PD-L1 ≥50%, ICI monotherapy represents a validated first-line alternative to chemoimmunotherapy. Three landmark phase 3 trials have demonstrated the superiority of ICI monotherapy over platinum-based chemotherapy: KEYNOTE-024 (pembrolizumab), IMpower110 (atezolizumab), and EMPOWER-Lung 1 (cemiplimab) with meaningful improvement in overall survival compared to chemotherapy (96–98).

A pooled analysis of KEYNOTE-010, -024, and -042 evaluated 132 patients aged ≥75 years with PD-L1 TPS ≥50% and showed that pembrolizumab monotherapy significantly improved OS compared with chemotherapy in this subgroup (HR 0.40), including among treatment-naïve patients (HR 0.41). Importantly, pembrolizumab was associated with substantially fewer treatment-related adverse events (TRAE) than chemotherapy, with grade ≥3 events occurring in 24.2% vs. 61.0% (99). Consistent with these findings, a Japanese prospective phase II study of first-line pembrolizumab in patients aged ≥75 years with PD-L1 TPS ≥50% (n=26; median age 79 years) reported a mPFS of 9.6 months and mOS of 21.6 months, with grade ≥3 TRAE in only 15.4% (100). Likewise, a multicenter retrospective study of 381 patients receiving first-line pembrolizumab for PD-L1 ≥50% NSCLC found no significant differences in time on treatment or OS between patients aged ≥70 and <70 years, with ECOG PS, rather than age, driving outcomes. However, patients aged ≥70 years were significantly less likely to receive second-line therapy after progression (101).

Regarding BrM, the evidence supporting ICI monotherapy in the PD-L1 ≥50% setting remains limited. KEYNOTE-024 excluded patients with untreated BrM, in the pembrolizumab arm 11.7% had treated baseline BrM. However, no dedicated CNS subgroup analysis has been reported in the final publication (96). IMpower110 similarly allowed only treated, asymptomatic CNS metastases and did not report BrM-specific outcomes (97). EMPOWER-Lung 1 enrolled patients with radiotherapy-treated, clinically stable BrM, 12% of the PD-L1 ≥50% population had previously treated symptomatic BrM. In this dedicated subgroup, cemiplimab improved OS (52.4m vs 20.7m), PFS (12.5m vs 5.3m), and quality of life compared with chemotherapy. Notably, the magnitude of benefit in patients with treated BrM was numerically greater than that observed in patients without BrM (98, 102). A phase 2 trial (N = 42) evaluated pembrolizumab in NSCLC patients with PD-L1 ≥1% and untreated or progressing asymptomatic brain metastases (5–20 mm), with a median age of 60 years, demonstrating an iORR of 29.7% and a 2-year OS of 34%, though patients were not stratified by PD-L1 expression, limiting the interpretation of these results.

These data provide the rationale for considering ICI monotherapy as a preferred strategy in older patients with high PD-L1 expression, particularly when the added toxicity of chemotherapy may not translate into meaningful survival benefit. Across all studies, ECOG performance status remains a robust independent predictor of poor prognosis and should be central to treatment decision-making in this population.

## Targeted therapy in elderly patients with actionable mutations

The ASCO Living Guideline (2026) and the ESMO/SIOG (International Society of Geriatric Oncology recommendations state that targeted therapy should be offered to older adults with oncogene-driven NSCLC, guided by a comprehensive GA and individualized decision based on overall health status, treatment goals, and patient preferences. Importantly, age alone should not preclude the use of targeted therapy in this population (103, 104).

## EGFR-mutant NSCLC

BrM occur in approximately a quarter (24.4%) of patients with EGFR-mutant NSCLC at the time of a diagnosis of advanced-stage disease, with a 5-year cumulative incidence as high as 52.9% (105). The three pivotal first-line trials with robust PFS, OS and CNS data are FLAURA (osimertinib monotherapy), FLAURA2 (osimertinib plus chemotherapy), and MARIPOSA (amivantamab-lazertinib).

The FLAURA trial (N = 556) compared osimertinib versus erlotinib or gefitinib in treatment-naïve EGFR-mutant advanced NSCLC (median age 64 years; range 26–85). Osimertinib demonstrated superior PFS (18.9 vs. 10.2 months) and OS (38.6 vs. 31.8 months; HR 0.80), however, in subgroup analyses, the OS benefit was not statistically significant among patients aged ≥65 years (106, 107). Although the study allowed patients with asymptomatic or stable CNS metastases, brain imaging was not mandated unless clinically indicated, likely underestimating the true prevalence of CNS disease, as only 36% had baseline brain imaging. Among 128 patients with measurable and non-measurable CNS lesions, CNS ORR was 66% versus 43%, and median CNS-PFS was not reached (95% CI, 16.5 months to NC) versus 13.9 months, favoring osimertinib. In the subgroup with measurable baseline BrM, CNS ORR was 91% versus 68%, again favoring the third-generation TKI (108).

The FLAURA2 trial (N = 557) addressed whether adding chemotherapy (cisplatin or carboplatin plus pemetrexed) to osimertinib could improve outcomes in patients with EGFR-mutant NSCLC (median age 61–62 years; range 26–85). The combination improved both PFS (25.5m vs. 16.7m) and OS (47.5 vs. 37.6 months), but was associated with a higher rate of grade ≥3 adverse events compared with osimertinib alone (70% vs 34%). Notably, in subgroup analysis, the OS benefit was not statistically significant for patients older than 65 years or for those without CNS involvement (9, 109). The CNS analysis in FLAURA2 is particularly robust, as all patients underwent mandatory brain imaging at baseline. Baseline CNS lesions were present in 42% of patients in the combination arm, including both measurable and non-measurable disease. Among patients with measurable CNS lesions (combination, n = 40; monotherapy, n = 38), CNS ORR was similar (88% vs. 87%), and CNS complete response rates favored the combination (48% vs. 16%). CNS-PFS was also longer with the combination, being not reached (95% CI, 23 months to NC) versus 17.3 months with monotherapy. In addition, 13 patients had leptomeningeal disease, and the median duration of treatment was longer with the combination (25.2 vs 12.0 months). When patients with both measurable and non-measurable CNS disease were considered together, CNS ORR was 73% versus 69%, and median CNS-PFS was 30.2 versus 27.6 months, both favoring the combination. At 36 months, OS among patients with brain metastases was 57% versus 40% (110).

The MARIPOSA trial (N = 1,074) evaluated a novel therapeutic strategy in first-line EGFR-mutant advanced NSCLC, combining a bispecific EGFR-MET antibody (amivantamab) with a third-generation EGFR TKI (lazertinib) versus osimertinib monotherapy. Median age was 63–64 years (range 25-88), with approximately 33% of patients aged 65–74 and 12% aged ≥75. The combination improved PFS (23.7 vs. 16.6 months; HR 0.70) and OS (HR 0.75; 3-year OS 60% vs. 51%), with similar ORR (around 85% in both arms). However, in subgroup analyses, neither PFS nor OS benefit reached statistical significance for patients aged ≥65 or ≥75 years, though both endpoints favored the combination in patients aged < 75 years (111, 112). Brain imaging was mandatory at enrollment (approximately 40% had baseline asymptomatic or stable brain metastases). Median intracranial PFS was 25.4 vs. 22.2 months (HR 0.79; 95% CI 0.61–1.02), with similar intracranial ORR (77–78%). At 36 months, 36% vs. 18% of patients remained alive and free of intracranial progression, suggesting durable intracranial benefit despite the non-significant HR. The combination therapy was associated with considerable toxicity, with 80% of patients experiencing at least one grade ≥3 adverse event (AE), compared with 52% in the monotherapy arm. Venous thromboembolism was reported in 40% of patients in the experimental arm. Notably, among patients aged ≥65 years, 85% experienced grade ≥3 adverse events versus 57% in the monotherapy arm, and fatal adverse events were also more frequent with the combination (15 vs 8 patients) (111).

An indirect comparison between FLAURA2 and MARIPOSA (2025) suggested that osimertinib + chemotherapy may be more effective in patients with BrM (PFS HR 0.63 and intracranial PFS HR 0.52, both favoring FLAURA 2), while no significant difference was seen in patients without brain metastases (113). Although these results are hypothesis-generating, they may still be relevant for therapeutic decision-making in older patients with BrM, particularly in light of the toxicity profile observed among older patients in MARIPOSA.

## Safety in older patients

The osimertinib (Tagrisso) FDA label provides age-stratified safety data. Among 1,813 patients treated with osimertinib monotherapy, 770 were ≥65 years and 207 were ≥75 years. Exploratory analysis from the FDA label demonstrates a higher incidence of grade ≥3 adverse reactions (43% vs. 33%) and more frequent dosage modifications (34% vs. 23%) in patients ≥65 compared to younger patients, though no overall differences in safety or effectiveness were observed. When combined with chemotherapy (FLAURA2), the disparity widens further, grade ≥3 adverse reactions occurred in 68% vs. 61%, with dosage modifications in 55% vs. 43% of older vs. younger patients, notably, this regimen did not include sufficient numbers of patients aged ≥65 to determine whether they respond differently from younger patients (114).

The amivantamab plus lazertinib FDA label reports no clinically meaningful differences in safety or efficacy between patients aged ≥65 and younger patients. However, as described above, grade ≥3 AEs were substantially more frequent with the combination, driven mainly by dermatologic toxicity, venous thromboembolism, and infusion-related reactions, which warrants careful consideration in the elderly population (115).

In older adults, more robust data are available for osimertinib monotherapy than for combination regimens. A Japanese real-world study (HOT2002) including 132 patients aged ≥75 years treated with first-line osimertinib reported a median PFS of 19.4 months, an ORR of 75.2%, and a median OS that was not reached (95% CI, 24.6 months to NR). Most patients had good functional status (85.6% ECOG PS 0–1), whereas 11.4% had PS 2 and 3% had PS 3–4. No significant difference in efficacy was observed between patients aged ≥80 and those aged <80 years, whereas PS ≥2 emerged as a poor prognostic factor. Pneumonitis occurred in 17.4% of patients, including grade ≥3 events in 9.1%, and more than two-thirds of pneumonitis-related discontinuations occurred within the first 3 months of treatment. Dose reduction was required in 40.9% of patients. No dedicate BrM analysis was performed (116). Complementing these real-world findings, the prospective phase II OPEN/TORG2040 trial provides data in a particularly vulnerable population, it enrolled 30 patients with EGFR-mutant NSCLC and poor PS (ECOG PS 2–4) to receive first-line osimertinib; median age was 75 years (range 41–92), and 60% had baseline BrM. The disease control rate was observed in 93.3%, and PS improvement in 63.3% of patients. Median PFS was 8.0 months, and mOS was 25.4 months. However, interstitial lung disease occurred in 20.0% of patients, and 26.7% experienced serious AE leading to treatment discontinuation, underscoring the need for close monitoring in this frail population (117). Beyond efficacy, cardiovascular toxicity warrants attention in older patients, a real-world study including 1,126 patients demonstrated a cardiotoxicity incidence of 4.7% with osimertinib, higher than reported in clinical trials. Advanced age, along with a history of heart failure and atrial fibrillation, were independent risk factors, however cardiac recovery was observed in 82.4% of affected patients (118).

## ALK-mutant NSCLC

ALK-rearranged NSCLC has a well-recognized CNS tropism, with a prevalence of BrM at diagnosis of approximately 35% and cumulative rates approaching 60% within 3 years (2, 119). Historically associated with poor prognosis, outcomes in this molecular subtype have been transformed by the evolution of targeted therapy. The phase III ALEX trial (N = 303, median age 58 years, range 25–88 in the experimental arm) was the first to demonstrate the superiority of a Alectinib, a next-generation ALK TKI, over crizotinib, with mPFS of 34.8 versus 10.9 months (HR 0.43) and a final mOS of 81.1 versus 54.2 months (HR 0.78; 95% CI 0.56-1.08). A total of 122 patients with asymptomatic BrM were enrolled in the ALEX trial, and alectinib demonstrated robust CNS activity, with an iORR of 81% versus 50% in patients with measurable CNS lesions (n = 43), and a CNS response rate of 59% among all patients with baseline CNS metastases in the alectinib arm. In the final OS analysis, mOS was notably improved in the BrM subgroup, 63.4 versus 30.9 months with alectinib versus crizotinib. In the primary PFS analysis, an age-subgroup forest plot (65 vs. ≥65 years) showed a consistent advantage with alectinib regardless of age, however, in the final OS analysis, the overall HR did not reach statistical significance, likely confounded by extensive crossover to next-generation ALK-TKIs in the crizotinib arm (120, 121). Exploratory safety analyses suggested that patients aged ≥65 years treated with alectinib experienced higher rates of serious adverse events (38% vs. 25%) and dose modifications (48% vs. 35%) than younger patients (122).

The other available option is brigatinib, which was evaluated in the phase III ALTA-1L trial (N = 275) versus crizotinib. The median age in the experimental arm was 58 years (range 27–86), and approximately 30% of patients in each arm had baseline BrM. At the final analysis, brigatinib achieved a mPFS of 24.0 versus 11.1 months in the Asian population and 24.7 versus 9.4 months in the non-Asian population, while mOS was not reached in either arm. Among patients with baseline BrM, the iORR was 62% versus 33% in the Asian population and 69% versus 3% in the non-Asian population, median iPFS was 43.3 months in the Asian population and not reached (30.8 months to not estimable) in the non-Asian population, both favoring brigatinib. In an age-specific analysis, no significant PFS benefit was observed in patients older than 65 years, and no dedicated OS analysis by age was provided (123, 124).

The phase III CROWN trial (N = 296) demonstrated unprecedented intracranial activity with lorlatinib in the first-line treatment of ALK-positive NSCLC. In the lorlatinib arm, the median age was 61 years (IQR 51–69), and 26% of patients had baseline CNS metastases (brain imaging was mandatory at study entry). After a median follow-up of 60.2 months, neither mPFS nor mOS was reached with lorlatinib. At 5 years, 60% of patients remained progression-free compared to 8% with crizotinib (HR 0.19). Intracranial efficacy was particularly striking. In the ITT population, the probability of being free from intracranial progression at 5 years was 92% with lorlatinib versus 21% with crizotinib (HR 0.06). Among patients with baseline BrM, this rate was 83% with lorlatinib, whereas all patients in the crizotinib arm had intracranial progression within 2 years (HR 0.03), in this subgroup, the iORR was 60% with lorlatinib versus 11% with crizotinib, including complete intracranial responses in 49% and 6% of patients, respectively. This benefit, however, comes at the cost of increased toxicity. The most common adverse events with lorlatinib included edema, weight gain, hypercholesterolemia, and hypertriglyceridemia. Grade 3–4 AE occurred in 77% of patients, compared to 57% with crizotinib. A particular concern in older patients is the neurocognitive side effect profile of lorlatinib, cognitive effects occurred in 21% of patients, mood effects in 16%, and visual disturbances in 18%, these events were typically grade 1–2 and generally reversible with dose modification. Dose reductions were required in 23% of patients. Notably, an exploratory analysis suggested that dose reduction within the first 16 weeks did not appear to compromise PFS or time to intracranial progression (10).

Different from EGFR-mutant NSCLC, dedicated real-world studies in older ALK-positive patients are scarce, largely because ALK rearrangements are more prevalent in younger populations. As no head-to-head trials have established the superiority of one next-generation ALK TKI over another, treatment choice remains nuanced. ESMO guidelines recommend lorlatinib as the preferred first-line option for patients with BrM (125), while ASCO guidelines similarly acknowledge lorlatinib’s superior CNS control but recommend that selection among first-line ALK TKIs should consider CNS risk, cardiac comorbidities, and the distinct adverse-effect profiles of each agent (103). In this context, a large network meta-analysis with 11 clinical trials across ALK-rearranged NSCLC offers some guidance, while lorlatinib achieved the longest PFS and highest ORR, alectinib demonstrated the most favorable OS and lowest incidence of grade ≥3 adverse events, with optimal efficacy in subgroups aged ≥65 years and those with PS 2, making it a particularly compelling option in the older population (126).

## KRAS-mutant NSCLC

KRAS mutations are the most common oncogenic driver in lung adenocarcinoma, occurring in approximately 20–30%, with the G12C variant representing roughly 40% of all KRAS-mutant tumors. BrM are common in this population, present in approximately 28% of patients at diagnosis and up to 40% over the disease course (127). The development of KRAS G12C inhibitors has introduced new therapeutic options with demonstrated intracranial activity.

In the phase III KRYSTAL-12 trial (N = 453), adagrasib was compared with docetaxel in patients with previously treated advanced/metastatic KRAS G12C-mutant NSCLC (median age 64 years, 47% aged ≥65 years, and 26% with baseline CNS metastases in the experimental arm). Adagrasib improved mPFS compared with docetaxel (5.5 vs. 3.8 months) and achieved a higher ORR (32% vs. 9%), with consistent PFS benefit across all subgroups, including older patients and baseline BrM. Among patients with measurable baseline BrM, the iORR was 40% with adagrasib versus 11% with docetaxel. OS data remain immature (128). In the phase III CodeBreaK 200 trial (N = 345), sotorasib was compared with docetaxel in patients with previously treated advanced/metastatic KRAS G12C-mutant NSCLC (median age 64 years, 46% aged ≥65 years; approximately 34% with CNS involvement in the experimental arm). Sotorasib demonstrated superior mPFS compared with docetaxel (5.6 vs. 4.5 months), without a significant OS benefit, with fewer treatment-related adverse events compared with docetaxel (33% vs. 40%) and prolonged median time to CNS progression in patients with baseline BrM (15.8 vs. 10.5 months) (129, 130).

Dedicated data in older and less fit patients come from the phase II ETOP ADEPPT trial, presented in abstract form which evaluated adagrasib in patients aged ≥70 years (ECOG PS 0–1) and in those with poor PS (ECOG PS 2). The confirmed ORR at 12 weeks was 31% in the older cohort and 18% in the ECOG PS 2 cohort; mPFS was 7.6 and 2.7 months, and mOS was 9.5 and 4.3 months, respectively. While awaiting full publication, these findings again suggest that performance status may be more important than chronological age (131). Regarding tolerability, a systematic review and meta-analysis presented in abstract format (2025 ASCO) comparing standard-dose sotorasib (960 mg) versus reduced starting doses found no statistically significant difference in ORR (32% vs. 26%) or PFS, while the standard dose was associated with dose reductions in 16% and discontinuation in 9%, While full publication is awaited, these findings suggest that reduced dosing may improve tolerability without significantly compromising efficacy, a consideration of particular importance in older patients susceptible to gastrointestinal and hepatic toxicities (132).

## Targeted therapy for other actionable drivers

MET exon 14 skipping mutations are found in roughly 3–4% of metastatic NSCLC cases, with brain metastases occurring at rates (20–40%) comparable to those seen in NSCLC lacking targetable driver alterations. Notably, patients with MET exon 14 skipping mutations are typically 70 years of age or older (133). In the phase II GEOMETRY mono-1 trial, the enrolled population was predominantly older, with a median age of 71.3 years; 85% of patients were aged ≥65 years, 29% were aged ≥75 to <85 years, and 4% were aged ≥85 years. Capmatinib showed meaningful systemic activity across treatment settings, achieving an ORR of 68% in treatment-naïve patients and 44% in previously treated patients, with mPFS of 12.5 and 5.5 months and mOS of 21.4 and 16.8 months, respectively. Intracranial activity was encouraging, with iORR observed in 57% of the 28 patients with BrM, regardless of prior intracranial radiotherapy (134). Similarly, in the phase II VISION trial, which enrolled 313 patients across the primary and confirmatory cohorts, the population was also predominantly older, with a median age of 72 years, and 57 patients had baseline BrM. At long-term follow-up, tepotinib achieved an ORR of 51.4%, with a mPFS of 11.2 months and a mOS of 19.6 months, with even greater benefit observed in treatment-naïve patients. Efficacy was maintained across age subgroups, with ORRs of 48.8% in patients aged <75 years and 39.7% in those aged ≥75 years. Intracranial activity was also notable, with an iORR of 66.7% among 15 patients with measurable BrM evaluable by RANO-BM criteria, most of whom had previously received brain radiotherapy (133). Both agents demonstrated a manageable safety profile, with peripheral edema and gastrointestinal events (nausea, vomiting) as the most frequent adverse effects (133, 134). Given that the majority of patients enrolled in both pivotal studies were older, these oral targeted therapies represent a particularly attractive treatment option for this subgroup of patients with this molecular alteration.

The hallmark toxicities of MET TKIs include peripheral edema, liver function abnormalities, elevated creatinine, and gastrointestinal toxicities, with a similar profile observed between tepotinib and capmatinib (133, 134). Real-world data are particularly relevant in this context, as almost 70% of all tepotinib adverse event reports came from the elderly population (135). Dose optimization is an emerging and clinically relevant strategy in this setting; a small real-world study demonstrated that 82% of patients (median age 76 years) required dose reduction, most commonly to 50% of the standard dose, with continued clinical benefit (136). Considering the higher prevalence of MET exon 14 skipping mutations in older patients, this is an area that deserves further dedicated investigation.

Mutations in the gene encoding human epidermal growth factor receptor 2 (HER2, also called ERBB2) drive approximately 2–4% of NSCLC and are associated with female sex, never-smoking history, and a higher incidence of brain metastases (47% at diagnosis) (137). Three FDA-approved HER2-targeted agents are now available: trastuzumab deruxtecan (T-DXd), zongertinib, and sevabertinib.

T-DXd was evaluated at two dose levels across the DESTINY-Lung trials. In the final analysis of DESTINY-Lung02 (n = 152; median age 59.4 years), the 5.4 mg/kg dose achieved an ORR of 50% and a median PFS of 10.0 months with a more favorable safety profile compared to the higher dose, with grade ≥3 adverse events 39.6% vs. 60.0% and drug-related ILD 14.9% vs. 32.0% respectively, most events being grade 1–2 (138). A pooled analysis of DESTINY-Lung01 and DESTINY-Lung02 demonstrated meaningful intracranial activity with an iORR of 50% and a complete response rate of 21% among patients with measurable BrM treated at 5.4 mg/kg, and median iDOR of 9.5 months (139). Real-world data from the TRACER/HERTras cohort (median age 62 years) further supported these findings, showing an iORR of 74.1%, including patients with active BrM (140).

Zongertinib, an oral irreversible HER2-selective TKI, achieved an ORR of 71% and a median PFS of 12.4 months in previously treated patients with HER2 tyrosine kinase domain (TKD) mutations (Beamion LUNG-1 cohort 1; median age 62 years, 43% aged ≥65 years), with similar efficacy observed across age groups and iORR of 64% among patients with stable or asymptomatic BrM. In the non-TKD mutations cohort, the ORR was lower at 30%. In the first-line setting cohort, restricted to patients with HER2 TKD mutations (median age 67 years), the ORR was 77%. Zongertinib also demonstrated activity after prior T-DXd (ORR 48%) (141, 142). The safety profile was favorable, grade ≥3 adverse events in 17%, no drug-related ILD, and a discontinuation rate of only 3%; diarrhea was the most frequent toxicity (56%), though mostly grade 1. Across all clinical studies, 47% of patients were aged ≥65 years and 13% were ≥75 years, with no differences in safety or effectiveness between age groups OS data are not yet available (143).

Another emerging option is sevabertinib, an oral, reversible HER2 TKI, demonstrated activity across multiple treatment settings in the phase I/II SOHO-01 trial, with more than 90% of patients harboring HER2 TKD mutations. ORRs were 64% in previously treated HER2-naïve patients (cohort D; median age 60 years), 71% in treatment-naïve patients (cohort F; median age 65 years), and 38% after prior HER2-targeted ADCs (cohort E; median age 65 years). Grade ≥3 adverse events occurred in 31%, with diarrhea as the most common toxicity, rash and paronychia were also frequent. Among patients aged ≥75 years, grade 3 diarrhea was more frequent compared to younger patients (23% vs. 14%). Overall, 43% were aged ≥65 years and 13% were ≥75 years, with no apparent differences in effectiveness by age group. Data from cohort G evaluating active brain metastases are pending publication (144, 145).

Oral TKIs such as zongertinib and sevabertinib are particularly attractive for older patients given their oral convenience, absence of ILD risk, low treatment discontinuation rates, and demonstrated intracranial activity. However, real-world data in older patients are currently lacking, as these agents are newly approved, and their tolerability and efficacy in this population remain to be prospectively assessed.

Beyond the molecular alterations discussed above, several other actionable driver mutations in NSCLC, including RET fusions (1–2%), ROS1 fusions (1–2%), BRAF V600E mutations (1–2%), and NTRK fusions (<1%), have effective targeted therapies available.

Patients with RET-mutant-NSCLC are known to have a high prevalence of BrM at diagnosis (32.2%) (119). In the phase I/II LIBRETTO-001 trial (N = 105), selpercatinib demonstrated substantial activity in previously treated patients, achieving an ORR of 64% and a median PFS of 16.5 months by independent review (146). In the first-line setting, the phase III LIBRETTO-431 trial (N = 261) compared selpercatinib with platinum-based chemotherapy with or without pembrolizumab. The median age in the selpercatinib arm was 61 years, and 37% of patients were aged ≥65 years. Selpercatinib achieved an ORR of 84% and significantly improved mPFS compared with the control arm (24.8 vs. 11.2 months). Among the 22% of patients with baseline BrM, the iORR was 82%, including complete responses in 35%. Although grade ≥3 AE were reported in 70% of patients, treatment discontinuation due to toxicity occurred in only 10%, the most common grade ≥3 AE were ALT/AST elevation, hypertension and QTc prolongation. OS data remain immature (147). Pralsetinib is another effective option in RET fusion-positive NSCLC, in treatment-naïve patients, it achieved an ORR of 78%, with a mPFS of 15 months and a mOS of 50.1 months, whereas in previously treated patients the ORR was 63% and mOS was 39.7 months. Among 15 patients with measurable BrM, the intracranial ORR was 53%. However, this efficacy must be balanced against a less favorable toxicity profile, as grade ≥3 adverse events occurred in 66% of patients (148). In addition, the ongoing phase III AcceleRET-Lung trial identified an imbalance in severe and fatal infections, including opportunistic infections, in the pralsetinib arm, which is of particular concern in older patients (103).

For ROS1-positive disease entrectinib, repotrectinib, and taletrectinib are all available first-line options with CNS penetration (149). Taletrectinib achieved an ORR of 88.8%, a mPFS of 45.6 months, and an iORR of 76.5% in TKI-naïve patients, with a safety profile characterized mainly by gastrointestinal toxicity and infrequent neurologic AE (150). Repotrectinib demonstrated an ORR of 79%, a mPFS of 35.7 months, and an iORR of 89% in TKI-naïve patients with measurable BrM, notably 83% of intracranial responses lasting at least 12 months. The most common AE were dizziness (58%), dysgeusia (50%), and paresthesias (30%) (151). Entrectinib, while effective (iORR 80% in treatment-naïve patients), carries a warning for CNS toxicity, including cognitive impairment and mood disorders, which may be of particular concern in older adults (152). Robust geriatric-specific data are limited for all ROS1 agents. Given its durable systemic and intracranial efficacy with a favorable safety profile, the ASCO guideline highlights taletrectinib as one of the preferred first-line options (103).

Patients with BRAF V600E-mutant NSCLC have a reported prevalence of BrM at diagnosis of approximately 26% (153). Recent data are increasing the support for the use of targeted therapy in the front line. In the phase II PHAROS trial, encorafenib plus binimetinib was evaluated in a relatively older population (median age 70 years), of whom 60% were treatment-naïve and only 8% had baseline BrM. The combination achieved an ORR of 75%, with a mPFS of 30.2 months and a mOS of 47.6 months in treatment-naïve patients, in previously treated patients the mPFS was 9.3 months with a mOS of 22.7 months (154). Dabrafenib and trametinib is another active option and was evaluated in a phase 2 trial with a median age of 65 years, including 36 treatment-naïve and 57 previously treated patients. Outcomes were broadly similar across treatment lines, with median PFS of approximately 10 months and median OS of around 17–18 months (155). However, the optimal first-line approach remains uncertain. In the multicenter retrospective FRONT-BRAF study, which included a relatively older population (median age 68 years; approximately 44% aged >70 years) and 48 patients with baseline BrM, first-line immune checkpoint inhibitors with or without chemotherapy were associated with longer OS than BRAF/MEK inhibition (40.9 vs. 25.2 months; HR 0.69), particularly among patients aged ≥70 years (HR 0.54) and those without BrM (156). Both dabrafenib plus trametinib and encorafenib plus binimetinib have shown favorable efficacy and manageable safety in both first-line and later-line settings, with common toxicities including pyrexia, skin toxicity, gastrointestinal adverse events, and fatigue. Thus, there is still no consensus regarding whether targeted therapy or immunotherapy should be preferred as the initial treatment strategy in this population (103).

NTRK gene fusions are rare oncogenic drivers in NSCLC, occurring in approximately 0.2–1% of cases. Three TRK inhibitors including entrectinib, larotrectinib, and repotrectinib are available as first-line options, with all three demonstrating CNS activity. In the updated integrated analysis of NTRK fusion-positive NSCLC (n = 51; median age 60 years, range 22–88), entrectinib achieved an ORR of 63%, a mPFS of 28.0 months, and a mOS of 41.5 months, among 14 patients with measurable BrM by BICR, the iORR was 64%, with a remarkable intracranial DOR of 55.7 months (157). In the updated larotrectinib lung cancer cohort (n = 32; median age 56 years, range 25–81; 12 with baseline CNS metastases), the ORR was 69%, with a median DoR of 34 months, a mPFS of 22 months, and a mOS of 41 months; among patients with baseline CNS metastases, the ORR was 67%–80% (158). In the TRIDENT-1 trial, repotrectinib demonstrated an ORR of 59% in TKI-naïve patients with a mPFS of 30.3 months, and retained activity in TKI-pretreated patients (ORR 48%) (159). Adverse events for all agents are predominantly dizziness, weight gain, fatigue, cognitive impairment, and transaminitis which is of particular concern in the older population (103). There is no consensus on the optimal sequencing of TRK inhibitors, although the NCCN guidelines include repotrectinib as an option after progression on entrectinib or larotrectinib if not previously given, and there are no specific data in older adults due to the rarity of this alteration (149).

## Conclusion

### Challenges

Older adults constitute a substantial proportion of patients with NSCLC, with more than half of new diagnoses occurring in individuals aged 65 years or older, and approximately 37% in those aged 75 years or older. This population often presents with a distinct clinical profile characterized by multimorbidity, polypharmacy, reduced physiologic reserve, and a high prevalence of baseline cognitive impairment. This vulnerability may be further compounded by the neurocognitive burden of brain metastases, which affect up to 50% of patients over their disease course depending on their molecular alterations.

Guidelines recommend that a comprehensive GA should be integrated into the oncologic decision-making process for all patients aged 65 and older with advanced NSCLC. While a full GA may not be feasible due to time or resource constraints, validated screening tools offer practical alternatives to identify patients who may benefit from a comprehensive GA or additional supports and interventions. Similarly, validated tools for predicting chemotherapy-related toxicity should be incorporated into treatment planning to guide therapy selection and dose optimization, thereby helping to balance efficacy against the risk of treatment-related harm in this vulnerable population.

Despite the rapid evolution of therapeutic strategies in advanced NSCLC, including combination chemoimmunotherapy regimens, dual immune checkpoint blockade, novel targeted agents such as bispecific antibodies, targeted therapies and treatment intensification approaches, older adults remain critically underrepresented in the pivotal clinical trials that define current standards of care. Across the landmark studies reviewed herein, patients aged ≥75 years typically represented fewer than 10–12% of enrolled populations, and age-specific subgroup analyses, when available, were consistently underpowered and exploratory. Notably, several pivotal trials failed to demonstrate statistically significant benefit in patients aged ≥65 or ≥75 years, raising important questions about the generalizability of intensified regimens to this population. This evidence gap is particularly pronounced at the intersection of advanced age and BrM, where the convergence of age-related vulnerabilities, neurocognitive risks related to both disease and treatment, and the need for CNS-directed therapies creates a uniquely complex clinical scenario that has been virtually unaddressed by prospective data. For patients requiring local therapy, MCC discussion including neurosurgery is vital to optimal treatment selection, with an expanding role for SRS and hippocampal-avoidance whole brain radiation to minimize cognitive toxicities.

### Future perspectives

Moving forward, dedicated prospective studies and registries focusing on older adults with advanced NSCLC and BrM are urgently needed. These studies should incorporate GA endpoints, neurocognitive outcomes, and patient-reported quality-of-life measures alongside traditional efficacy endpoints. Until such data become available, treatment decisions for older patients with BrM must rely on individualized risk–benefit assessments that integrate geriatric evaluation, functional status, molecular profiling, patient preferences, and the cautious extrapolation of existing evidence, recognizing that the current therapeutic landscape was largely built on data from younger and fitter populations.
